# Effectiveness of VR-based cognitive training and games on cognitive rehabilitation in patients with MCI: a systematic review and meta-analysis

**DOI:** 10.3389/fneur.2025.1691344

**Published:** 2025-11-07

**Authors:** Peiming Yuan, Jiaxi Chen, Dianhui Peng, Qian Yang, Bin Liu, Chunxia Lu

**Affiliations:** College of Physical Education, Hunan Normal University, Changsha, China

**Keywords:** virtual reality, VR cognitive training, mild cognitive impairment, cognitive rehabilitation, games, meta-analysis

## Abstract

**Background:**

Mild Cognitive Impairment (MCI) represents a prodromal dementia stage marked by cognitive decline without functional impairment. Given limited drug efficacy and global aging, non-pharmacological interventions are urgently needed. Virtual reality (VR) enables immersive cognitive rehabilitation, yet evidence remains inconsistent due to divergent intervention approaches (training vs. gaming) and technical parameters like immersion level.

**Objective:**

This systematic review and meta-analysis synthesized evidence from randomized controlled trials (RCTs) to evaluate the efficacy of VR-based cognitive training and gaming interventions on cognitive function in older adults with MCI and to investigate the moderating role of immersion level.

**Methods:**

We systematically searched four electronic databases (PubMed, Web of Science, Embase, Scopus) from inception to July 20, 2025, for RCTs investigating VR interventions (cognitive training or games) in individuals aged ≥ 55 years diagnosed with MCI. Two independent reviewers performed study selection, data extraction (including intervention characteristics, implementation details, and behavior change techniques), and risk-of-bias assessment using the Cochrane Risk of Bias tool (RevMan 5.4.1). Standardized mean differences (Hedges’s g) with 95% confidence intervals (CI) were pooled using random-effects models in Stata 18.0. Heterogeneity was quantified using *I^2^*. Publication bias was assessed via funnel plots and Egger’s test. Pre-specified meta-regression explored immersion level as a potential moderator. The certainty of evidence was assessed using the Grading of Recommendations Assessment, Development and Evaluation (GRADE) system.

**Results:**

Of the 2,486 articles retrieved in total, 11 studies were included in the analysis. VR demonstrated a statistically significant improvement in the efficacy of cognitive rehabilitation among patients with MCI (Hedges’s g = 0.6, 95% *CI*: 0.29 to 0.90, *p* < 0.05). Specifically, VR-based games (Hedges’s g = 0.68, 95% *CI*: 0.12 to 1.24, *p =* 0.02) showed greater advantages in improving cognitive impairments compared to VR-based cognitive training (Hedges’s g = 0.52, 95% *CI*: 0.15 to 0.89, *p* = 0.05). The immersive level of VR interventions emerged as a significant moderator of heterogeneity across the included studies. Based on the GRADE criteria, the quality of evidence for the efficacy of VR-based interventions on cognitive function in individuals with MCI is moderate. A stratified analysis by intervention type showed that VR cognitive training is supported by moderate-certainty evidence, while evidence for VR games is of low certainty.

**Conclusion:**

VR-based interventions, including cognitive training and games, effectively improve cognitive function in MCI patients, with VR games showing a trend toward greater efficacy. Immersion level critically influences therapeutic outcomes, requiring optimized sensory integration while accommodating individual tolerance. These findings support supervised clinical VR training alongside engaging home-based protocols to enhance adherence. Future development of standardized immersion adjustment and personalized guidelines will advance utility across care settings.

## 1 Introduction

Mild cognitive impairment (MCI), defined as a level of cognitive ability that is lower than would be expected for their age and educational level, occurring between normal aging and dementia (1). Individuals diagnosed with MCI are at a significantly higher risk of progressing to dementia, with a mean annual conversion rate of approximately 10%, compared to the annual incidence of 1–2% in the general population (2, 3). Treatments can be implemented to slow down the advancement of dementia during the preclinical phase (4), making the identification of effective therapeutic strategies to delay or prevent the progression to dementia of utmost importance.

The increasing prevalence of MCI, driven by the aging global population, there are few medications or dietary therapy that can improve cognitive function or slow MCI progression, non-pharmacological treatments have received attention (3, 5). One of the non-pharmacological treatments is the use of virtual reality (VR) technology, which is currently employed in the control and treatment of various diseases (6). It helps the user create a real sense of presence and immersion in the virtual world through multiple sensory stimuli (visual, auditory, tactile, and olfactory) while also functioning by distracting them within that virtual and simulated environment (7, 8). Virtual reality technology demonstrates two principal manifestations in cognitive rehabilitation: VR-based cognitive training and VR-based games. This fundamental distinction reflects divergent methodological approaches to cognitive enhancement, each characterized by unique mechanisms of engagement and therapeutic delivery. In cognitive training, VR serves as a targeted intervention for specific cognitive domains—such as memory and attention—through repetitive, goal-oriented tasks (9). These projects may incorporate elements of “serious games” to increase engagement, but their main focus is on therapeutic training. For instance, integrating VR technology into routine training creates immersive experiences for individuals with mild cognitive impairment (9, 10). In contrast, VR games emphasize immersive narratives, exploration, and compelling gameplay (11, 12). Cognitive challenges are naturally embedded within story-driven objectives—such as solving puzzles or completing simulated missions (13). This approach prioritizes intrinsic motivation, presence, and enjoyment, facilitating cognitive exercise within ecologically rich environments (14). While both paradigms share the ultimate goal of cognitive enhancement, their differing design philosophies and engagement paradigms may yield differential outcomes in therapeutic efficacy, adherence patterns, and cognitive benefit profiles (15, 16), thereby directly informing the precision design of future VR interventions.

Several trials have investigated the impact of VR on older adults with MCI, but the findings have been inconclusive. For example, studies by Baldimtsi et al. (17) and Park et al. (10) revealed a significant effect of VR on general cognitive abilities. However, the findings from Park et al. (18) showed that a 12-week, culture-based VR training program did not improve general cognitive abilities and did not show significant differences in scores on the Mini-Mental Status Examination (MMSE). Also, it is challenging to make definitive conclusions about the effectiveness of interventions because of variations VR intervention content (cognitive training or games). In a study conducted by Yang et al. (16), daily life-based VR training games (making juice, shooting crows, finding the number of fireworks, and memorizing objects in the house) were found to positively affect general cognitive performance. However, in a study by Kang et al. (15), while the VR group participants received multidomain and neuropsychologist-assisted cognitive training, no significant differences in general cognitive performance were observed when compared to other groups or baseline measurements. To the best of our knowledge, although the use of VR technology to improve cognitive function is increasing (19), the impact of VR-based cognitive training and games on the cognitive rehabilitation of patients with MCI remains controversial (10, 14).

In conclusion, while existing studies have demonstrated the potential value of VR-based cognitive training, current evidence regarding its efficacy in MCI remains limited by methodological constraints. Notably, there is a scarcity of systematic reviews or meta-analyses specifically examining the cognitive rehabilitation effects of VR-based training and gaming interventions in the MCI population. To address this gap, this study conducted a comprehensive meta-analysis to quantitatively synthesize existing evidence and evaluate the therapeutic potential of VR interventions for cognitive rehabilitation in individuals with MCI.

## 2 Materials and methods

### 2.1 Search strategy

Studies were identified by searching web-based databases with support and consultation provided by institutional librarians. Four databases were searched (PubMed, Embase, Web of Science, Scopus) by combining keywords. To include studies reflecting the latest advancements in VR technology and methodologies, the focus was on literature published from January 2013 to July 2025. The year 2013 was chosen to ensure consistency in the technological sophistication and usability standards of VR interventions, as VR technology and its applications in cognitive rehabilitation have rapidly evolved over the past decade (20). Keywords and search strategies are included: (“Virtual Reality” OR “VR” OR “virtual environment” OR “Virtual Reality Training” OR “VR cognitive training” OR “virtual game” OR “Game” OR “Gaming” OR “video games”) AND (“Mild Cognitive Impairment” OR “MCI” OR “Cognitive Dysfunction” OR “Cognitive Disorder” OR “Cognitive Impairment” OR “cognitive decline”) AND (“treatment” OR “intervention” OR “rehabilitation” OR “therapy” OR “training”).

### 2.2 Selection criteria

#### 2.2.1 Inclusion criteria

The inclusion criteria were defined with the PICOS approach: (i) Studies concerning older adults (aged ≥ 55 years) with a confirmed diagnosis of MCI by neurologic examination or neuropsychological assessment were included. The diagnosis was typically operationalized through standardized cognitive cut-offs, most commonly a Mini-Mental State Examination (MMSE) score of 24–27 or a Montreal Cognitive Assessment (MoCA) score of 18–26, to define the presence of objective cognitive impairment while excluding frank dementia (21, 22); (ii) Intervention: VR-based cognitive training and gaming; (iii) Controls: Studies with any type of control group were included (inactive controls include educational programs or no intervention; active controls include traditional rehabilitation or any other type of physical activity, physical-cognitive co-training, or video games without VR components); (iv) Outcome: Overall cognitive function; (v) Study design: randomized controlled trials (RCTs); (vi) Additional: Published in English; full-text available.

#### 2.2.2 Exclusion criteria

The exclusion criteria were as follows:

I No specific identification of cognitive impairment: Studies where VR was not used in the intervention group, or VR was used in the control group, were excluded.
II Cognitive impairment caused by other conditions: Studies where cognitive impairment was attributed to other medical conditions, such as stroke, cerebral infarction, traumatic brain injury, or other neurological disorders, were excluded. This ensures that the cognitive impairment under study is specifically related to MCI and not secondary to other health issues.
III Materials such as books, book chapters, letters to the editor, and conference abstracts were excluded from the analysis.

### 2.3 Study selection and data extraction

The article search and selection process were reviewed through the title and abstract of searched articles after the primary database search and, in the full review, two authors finally selected the articles by considering the eligibility criteria. This process was performed using a preferred reporting items for systematic reviews and meta-analysis (PRISMA) flow chart (23). Data were extracted by 2 researchers (PY and DP) and cross-checked by a third researcher (JC). The data extraction form encompassed various fields, including the author, published year, country, study design, sample size (male/female), mean age, intervention in treatment group, intervention in control group, duration of the session and the follow-up period and outcome characteristics was extracted.

### 2.4 Classification of immersion level

Based on specific technical specifications—including stereoscopy, 3/6-DOF tracking, natural interaction paradigms, and advanced features such as haptic feedback—we established operational criteria to classify immersion into three distinct levels: Low, Moderate, and High (24, 25). Details in Table 1.

**Table 1:** Operational criteria for VR immersion levels.

Degree	Low immersion	Medium immersion	High immersion
Core systems and display devices	Non-immersive systems rely on standard 2D displays—including desktop monitors, television screens, or tablets—that lack stereoscopic vision and multi-sensory depth cues. These configurations offer limited perceptual engagement and a restricted field of view, preventing a truly immersive user experience.	Semi-immersive systems utilize head-mounted displays (HMDs) to deliver stereoscopic 3D visuals while isolating users from their physical environment.	Fully immersive systems employ advanced head-mounted displays that deliver high-resolution, wide-field-of-view stereoscopic 3D vision, creating profound visual encapsulation.
Tracking degrees of freedom	These systems provide no spatial tracking or only offer basic controller-based input tracking.	These systems provide 3-DOF tracking, capturing only rotational head movements (pitch, yaw, and roll).	These systems support full 6-DOF tracking, enabling simultaneous monitoring of positional movements (forward/backward, left/right, up/down) and rotational orientation for both the head and controllers.
Interaction modality and naturalness	These systems employ abstract, symbolic interaction through conventional input devices such as mice, keyboards, touchscreens, or standard game controllers.	These systems incorporate basic motion controllers that translate hand gestures—such as pointing and clicking—into virtual interactions, though with constrained precision and limited naturalism.	These systems implement natural interaction paradigms—such as 6-DOF motion controllers, hand tracking, or full-body tracking—enabling intuitive object manipulation that closely replicates real-world interactions with high fidelity.
Sensory involvement and feedback	Sensory engagement is minimal in these systems, primarily limited to visual and basic auditory feedback, resulting in a weak sense of presence.	These systems achieve moderate sensory engagement through stereoscopic vision and head motion tracking, which significantly enhance presence. Basic haptic feedback—such as controller vibration—may also be incorporated.	These systems deliver peak sensory engagement by integrating multi-sensory stimulation—including spatialized audio and advanced haptic feedback—to create deeply compelling and highly realistic experiences.

### 2.5 Risk of bias and GRADE assessment

The risk of bias assessment was conducted using the risk of bias tool from Rev. Man 5.4.1 (26). This tool evaluates seven aspects: random sequence generation, allocation concealment, blinding of participants and personnel, blinding of outcome assessment, incomplete outcome data, selective reporting, and other biases. Each aspect was rated by the researchers as high risk (−), low risk (+), or uncertain risk (?). In cases of disagreement on the ratings, a consultation process was implemented to reach a consensus. The certainty of evidence for each outcome was rated using the GRADE approach, which evaluates five key domains: risk of bias, inconsistency, indirectness, imprecision, and publication bias (27, 28). The evaluation process also incorporated an assessment of factors that could potentially upgrade the certainty of the evidence, such as a large magnitude of the effect estimate or evidence of a dose–response gradient. Following this comprehensive appraisal, the overall certainty of evidence for each outcome was categorized as high, moderate, low, or very low.

### 2.6 Statistical analysis

The included studies were synthesized and analyzed using Stata version 18.0 software. Statistical heterogeneity, effect size, meta-regression, and publication bias were analyzed. Hedge’s g was used to calculate and interpret the effect size. For calculation and analysis of results, mean, standard deviation, and number of subjects were used as values. The heterogeneity was quantitatively determined by I2, where I2 values of < 25, 26–74, and > 75% represented small, moderate, and large levels of heterogeneity, respectively. Fixed-effects models were applied when heterogeneity was graded as small, whereas random-effects models were utilized for moderate or large heterogeneity (29). Publication bias refers to an error in which research results are published or not published depending on the characteristics or direction of research results. If a distorted sample of studies is included in a meta-analysis, the overall size of the analysis result can be said to be a distorted result (30). To confirm this tendency, it was reviewed and presented through a funnel plot and Egger’s regression test (31). In addition, meta regression was used to assess the sources of heterogeneity in the included studies.

## 3 Results

### 3.1 Study selection

**Figure 1:**
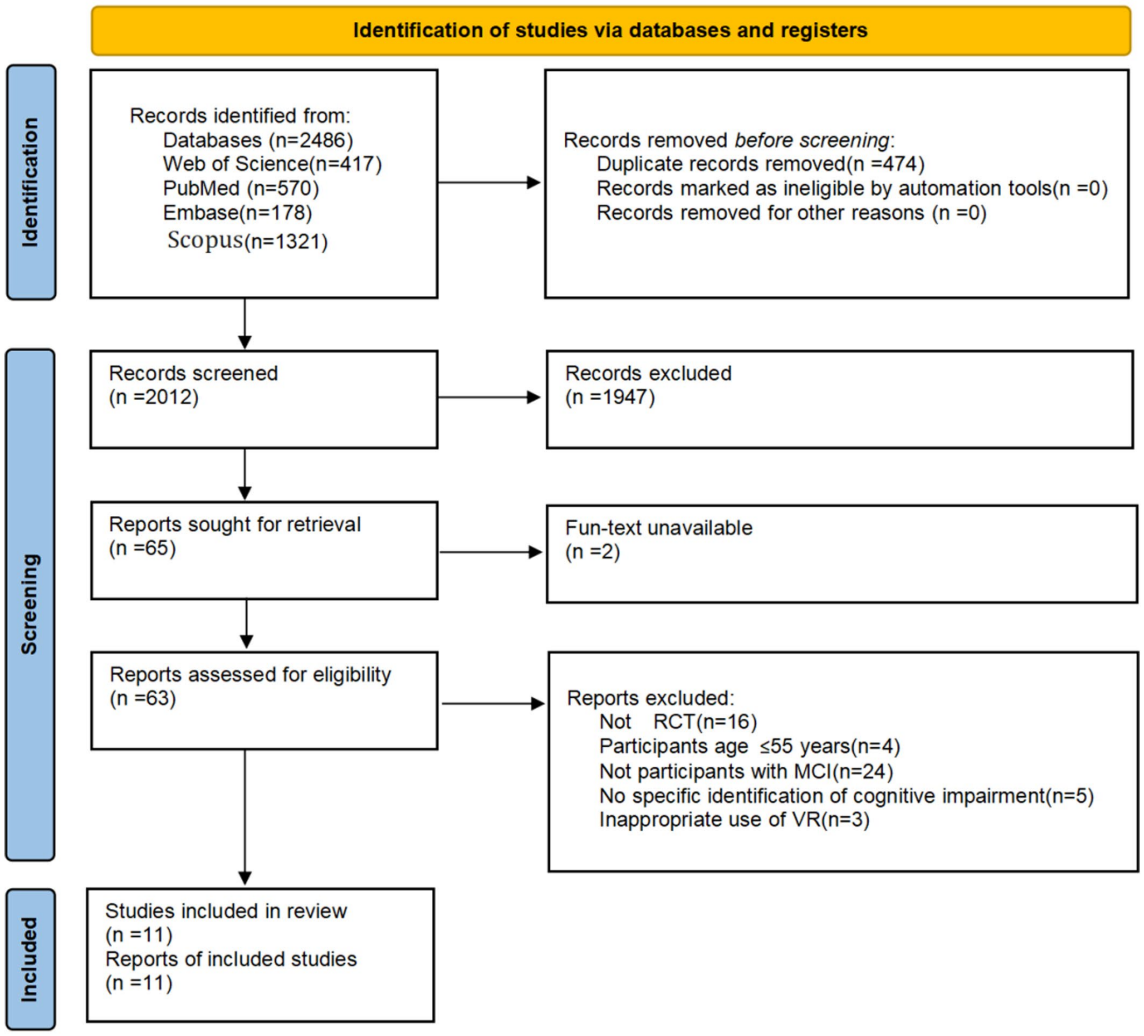
PRISMA (Preferred Reporting Items for Systematic Reviews and Meta-Analyses) flow diagram. MCI, mild cognitive impairment; RCT, randomized controlled trial; VR, virtual reality.

A total of 2,486 papers were identified using the four databases, of which 474 were duplicates. Each of these studies underwent a rigorous and meticulous review, during which inclusion and exclusion criteria were carefully applied. The assessment process involved a thorough examination of the methodologies, results, and relevance to the research focus. After this meticulous screening, 11 articles that met the inclusion criteria were finally selected. The detailed process of this screening is as follows Figure 1.

### 3.2 Characteristics of included articles

A total of 11 studies were included, among which 8 were conducted in South Korea, and the remaining 3 were carried out in China, Turkey, and Ecuador. All included studies focused on elderly individuals with mild cognitive impairment (MCI). Regarding interventions for cognitive rehabilitation in MCI patients, 6 studies adopted VR-based cognitive training, such as spatial cognitive training, simulated shopping activities, and virtual kayaking paddling exercises; the other 5 studies utilized VR-based games, including Seek a Song of Our Own, Fireworks Party, and Boxing Trainer. In one of the articles, no intervention was implemented in the control group, while other studies applied interventions such as conventional training or a combination of cognitive training and physical activities. The characteristics of the included articles were presented in Table 2. This study further establishes a systematic classification of immersion levels across all included studies, with clearly defined criteria based on specific hardware capabilities and interactive features. The detailed classification framework is presented in Table 3.

**Table 2:** Characteristics of the included articles.

Author, Year	Country	Study Design	Sample size (M/F)	Age (M ± SD)	Intervention in treatment group	Intervention in control group	Duration of the session and the follow-up period	Outcome
Park et al. (36)	Korea	RCT (single-blind)	EG: 28(12/16) CG: 28(11/17)	EG: 71.93 ± 3.11 CG: 72.04 ± 2.42	Virtual reality space cognitive training	No interference is accepted	56 sessions, 45 min/session, 3 days/week 8 weeks	WAIS-BDT
Kang et al. (15)	Korea	RCT	EG: 23(6/17) CG: 18(6/12)	EG: 75.48 ± 4.67 CG: 73.28 ± 6.96	Neuropsychologist-guide-d immersive VR cognitive training.	Usual therapy: pharmacotherapy	Approximately 20–30 min for each session, twice a week, for 1 month	MMSE
Buele et al. (9)	Ecuador	RCT (single-blind)	EG: 17(7/10) CG: 17(4/13)	EG:75.14 ± 5.76 CG:77.35 ± 6.75	VR kitchen search cognitive training.	Non-VR cognitive training task.	6-week intervention (a total of twelve 40-min sessions)	MoCA
Park J. S. et al. (10)	Korea	RCT	EG:18(10/8) CG: 17(7/10)	EG: 75.8 ± 8.5 CG: 77.2 ± 7.2	MOTOCOG®system	Tabletop activities	30 min per day, 5 days/week, for 6 weeks	MoCA
Choi et al. (33)	Korea	RCT	EG: 30(5/25) CG: 30(4/26)	EG: 77.27 ± 4.37 CG: 75.37 ± 3.97	Virtual kayak paddling exercise	Home exercises	60 min per day, 22 days/week, for 6 weeks	MoCA
Liao et al. (34)	China	RCT (single-blind)	EG: 18(7/11) CG: 16(4/12)	EG: 75.5 ± 5.2 CG: 73.1 ± 6.8	VR daily activities; Cognitive tasks	Combined Physical and Cognitive Training	60 min per day, 3 days/week, for 12 weeks	MoCA
Torpil et al. (37)	Turkey	RCT (single-blind)	EG: 30(11/19) CG: 31(14/17)	EG: 70.12 ± 2.57 CG: 70.30 ± 2.73	Four games (Boxing Trainer, Jet Run, Superkick, Air Challenge)	LOTCA-G cognitive domain intervention	45 min per day, 2 days/week, for 12 weeks	LOTCA-G
Thapa et al. (13)	Korea	RCT	EG: 34(6/28) CG: 34(10/24)	EG: 72.6 ± 5.4 CG: 72.7 ± 5.6	Four VR training games	An educational program focusing on overall healthcare	100 min per day, 3 days/week, for 8 weeks	MMSE
Lim et al. (35)	Korea	RCT (single-blind)	EG: 12(3/9) CG: 12(4/8)	EG: 75.42 ± 5.74 CG: 73.33 ± 17.52	Brain Talk™ home-based Serious game	Performing daily tasks	30 min per day, 3 days/week, for 4 weeks	MoCA
Yang et al. (16)	Korea	RCT	EG: 33(13/20) CG: 33(6/27)	EG: 72.5 ± 5.0 CG: 72.6 ± 5.6	Targeted cognitive games.	Health education seminars on geriatric nutrition and exercise.	100 min per day, 3 days/week, for 8 weeks	MMSE
Park J. H. et al. (18)	Korea	RCT	EG: 10(3/7) CG: 11(4/7)	EG: 71.80 ± 6.61 CG: 69.45 ± 7.45	Six VR Game Training Programs	Maintain normal daily activities	30 min per day, 2 days/week, for 3 months	MMSE

**Table 3:** Classification of immersion level in included studies.

Study (First Author, Year)	Assigned immersion level	Rationale for classification
Buele (9)	High	Utilized a fully immersive HMD with 6-DOF tracking, wireless controllers enabling natural interaction, and a fully enclosed interactive virtual environment.
Choi (33)	Low	The system employed a large projection screen instead of a head-mounted display, delivering a “virtual reality” experience more akin to immersive video viewing than interactive simulation.
Kang (15)	High	The study explicitly reported using an Oculus Rift CV1 head-mounted display with Oculus Touch controllers. As a high-end PC-VR system, the Oculus Rift provides 6-DOF positional tracking and enables natural interaction, establishing a fully immersive 3D virtual environment.
Liao (34)	High	The study utilized a fully immersive HTC VIVE head-mounted display with room-scale 6-DOF tracking, wireless controllers supporting natural interaction, and a complex interactive virtual environment based on activities of daily living.
Lim (35)	Low	The study was described as a “home-based serious game on a tablet computer,” with the intervention entirely delivered on a 2D flat screen. Participants interacted via touchscreen, without using a head-mounted display or possessing spatial tracking capabilities.
Park (36)	Low	The study utilized a desktop computer running a Unity program with joystick-controlled navigation. No head-mounted display was employed, and multi-sensory feedback was absent, resulting in a screen-based two-dimensional interactive experience.
Park J. H. (18)	High	The study employed an HTC Vive head-mounted display featuring a 2,160 × 1,200 resolution, 90 Hz refresh rate, and 110-degree field of view. The system supported 6-degree-of-freedom tracking and bimanual controller interaction, delivering fully immersive visual and auditory experiences.
Park J. S. (10)	Middle	The study utilized a PC-driven commercial HTC Vive head-mounted display, delivering high-resolution stereoscopic vision, approximately 110-degree field of view, 90 Hz refresh rate, and room-scale 6-degree-of-freedom tracking. Interaction was implemented through standard VR controllers. Although the tracking precision was high, the interaction modality remained conventional, with no mention of natural hand interaction or haptic feedback beyond standard vibration.
Thapa (13)	High	The study employed a commercial all-in-one head-mounted display (Oculus Quest), providing first-person perspective, stereoscopic vision, and 6-degree-of-freedom head tracking. Interactions were implemented through standard wireless VR controllers.
Torpil (37)	Low	The study utilized Microsoft Kinect for PC, explicitly described as operating “without immersion,” with visual content displayed on a 65-inch flat-panel screen. Participants stood before the television and controlled interactions through body movements. The setup lacked a head-mounted display and multi-sensory immersion capabilities.
Yang (16)	High	The study employed an Oculus Quest head-mounted display paired with two wireless hand controllers, delivering a fully immersive VR experience. The headset provided complete visual isolation and environmental occlusion.

### 3.3 Assessment of methodological quality

**Figure 2:**
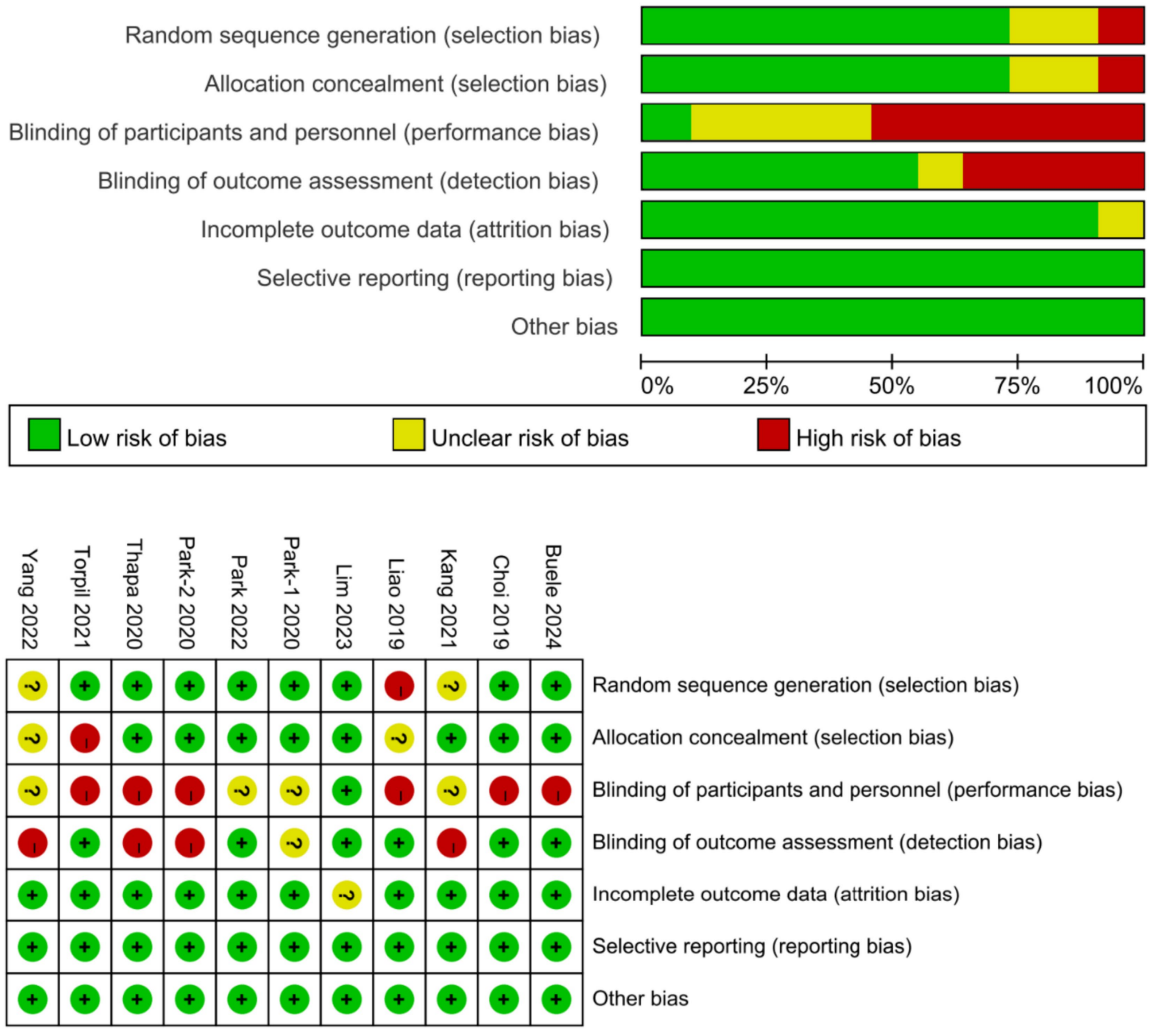
Risk of bias summary [Park et al. (36); Kang et al. (15); Buele et al. (9); Park et al. (10); Choi et al. (33); Liao et al. (34); Torpil et al. (37); Thapa et al. (13); Lim et al. (35); Yang et al. (16); Park et al. (18)].

The results of risk of bias assessment were as follows: random sequence generation (low: 8, uncertain: 2, high: 1), allocation concealment (low: 8, uncertain: 2, high: 1), blinding of participants and personnel (low: 1, uncertain: 4, high: 6), blinding of outcome assessment (low: 6, uncertain: 1, high: 4), incomplete outcome data (low: 10, uncertain: 1), selective reporting (low: 11), and other biases (low: 11). For other biases, items such as lack of sample size calculations, differences in baseline characteristics, and lack of study protocol registration were assessed as uncertain or high (32) (Figure 2).

**Figure 3:**
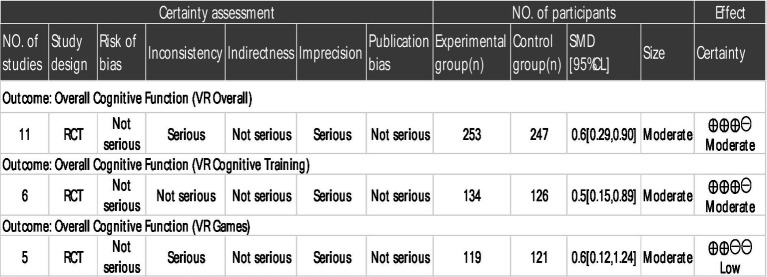
GRADE level of evidence rating scale for indicators of consequences.

According to the GRADE assessment, the certainty of evidence regarding the effect of VR interventions on overall cognitive function in patients with MCI was rated as moderate. This judgment was based on a balance of downgrading and upgrading factors. The evidence was downgraded due to substantial heterogeneity (*I^2^* = 64.69%) and imprecision resulting from a limited sample size and wide confidence intervals. However, it was upgraded based on a significant dose–response relationship identified in the meta-regression analysis, wherein a higher level of VR immersion was positively correlated with greater cognitive improvement (*β* = 0.834, *p* < 0.05). In subgroup analyses, the certainty of evidence was moderate for VR-based cognitive training but low for VR-based gaming. The latter was further downgraded to low certainty within its subgroup, primarily owing to considerable heterogeneity and more severe imprecision (as indicated by extremely wide confidence intervals; Figure 3).

### 3.4 Meta-analysis results

#### 3.4.1 The effect size on cognitive rehabilitation

**Figure 4:**
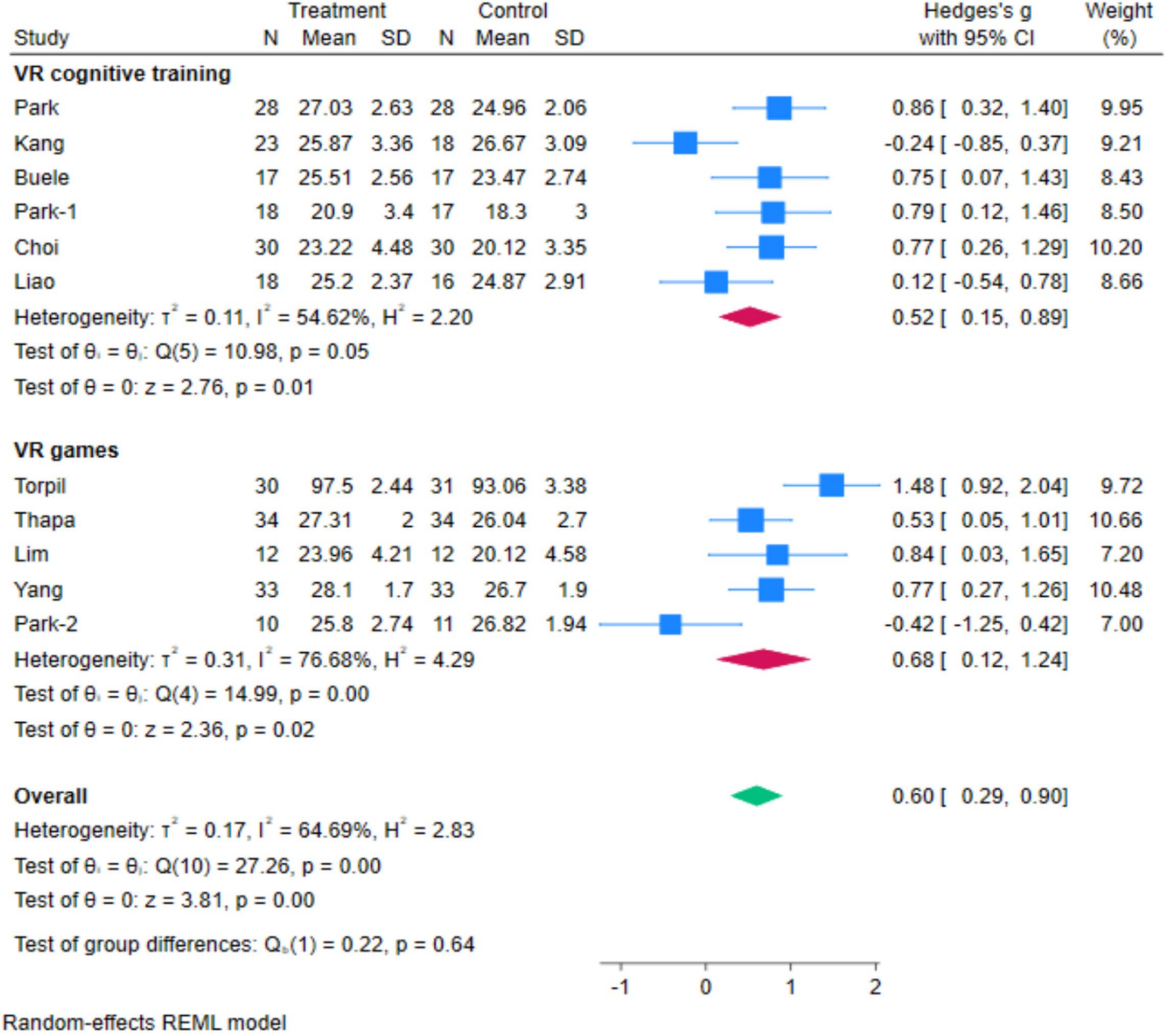
The forest map.

A total of 11 studies involving 500 MCI patients reported on the effects of VR technology based interventions on cognitive rehabilitation at post intervention time points (range 4 ~ 12 weeks) compared with conventional control conditions (Figure 4) (9, 10, 13, 15, 16, 18, 33–37).

The I^2^ between the included studies was >50%, thus a random-effects model was employed to assess the effect size. The research indicates that VR-based therapy has a significant positive impact on cognitive rehabilitation in individuals with MCI (Hedges’s g = 0.6, 95% *CI* 0.29 to 0.90, *p* < 0.05). The highest and lowest effect sizes were related to the study of Torpil (37) and Liao (34), respectively (Figure 4).

Based on Cohen’s d standardized effect size, this effect size is medium (38). Also, VR games (Hedges’s g = 0.68, 95% *CI*: 0.12 to 1.24, *p* = 0.02) have been demonstrated to improve cognitive disorders more effectively than cognitive training (Hedges’s g = 0.52, 95% CI: 0.15 to 0.89, *p* = 0.05).

#### 3.4.2 Publication bias

**Figure 5:**
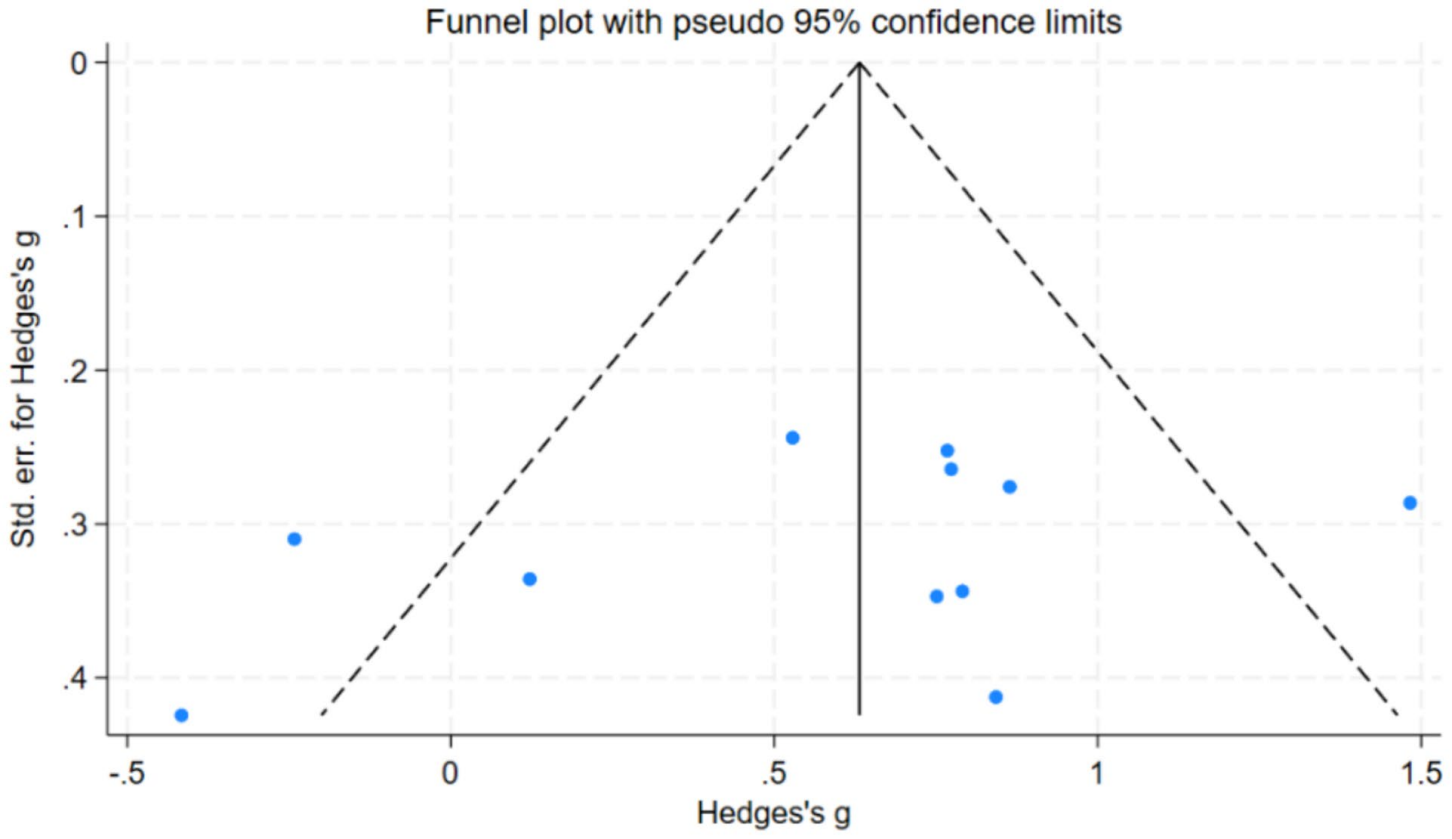
Publication bias of the included articles.

The funnel plot (Figure 5) illustrates the absence of publication bias in the studies. Moreover, the result of the Egger’s regression test was (*t* = −1.07, *p* = 0.31). This shows there is no publication bias.

#### 3.4.3 Meta-regression analysis

Meta-regression analysis identified the level of VR immersion as a statistically significant and positive moderator of cognitive improvement (coefficient *β* = 0.834, 95% *CI*: 0.211 to 1.457, *p* < 0.05), indicating that each unit increase in immersion level (e.g., from “low” to “medium” or “medium” to “high”) was associated with an average 0.834 increase in effect size (Table 4). In contrast, the analysis revealed that while intervention duration (*p* = 0.089) and blinding implementation (*p* = 0.072) did not reach conventional statistical significance thresholds, their coefficient estimates and confidence intervals suggested meaningful effect sizes approaching significance. These findings indicate potential moderating trends that merit examination in future studies with larger sample sizes. Other covariates, including VR intervention type and outcome measurement characteristics, demonstrated no appreciable relationship with effect size (*p* > 0.15), supporting their exclusion as substantive moderators in the current data set (Table 4).

**Table 4:** Effect sizes for separate meta-analyses on moderator variables.

Covariates	Coefficient	Standard error	95% CI	*p*
Duration of intervention	−0.446	0.212	(−0.992, 0.099)	0.089
Blind implementation	0.891	0.392	(−0.118, 1.900)	0.072
Immersive level	0.834	0.242	(0.211, 1.457)	0.018
Type of VR intervention	0.361	0.216	(−0.193, 0.915)	0.154
Result measure characteristics	0.075	0.127	(−0.251, 0.401)	0.581

## 4 Discussion

The present systematic review and meta-analysis specifically focuses on the efficacy of VR-based cognitive training and games in patients with MCI, providing targeted insights into this critical transitional stage between normal aging and dementia. The findings indicate that both VR-based cognitive training and games exert significant positive effects on cognitive rehabilitation in MCI patients, with a medium overall effect size (Hedges’s g = 0.60, *p* < 0.05). Subgroup analysis further reveals that VR games (Hedges’s g = 0.68) yield a slightly larger effect size than VR cognitive training (Hedges’s g = 0.52), though the difference is not statistically significant (*p =* 0.64). Additionally, meta-regression identifies VR immersion level as a key moderator of intervention efficacy, highlighting its potential role in optimizing therapeutic outcomes.

The therapeutic benefits of VR-based cognitive rehabilitation in MCI can be attributed to the condition’s distinctive neuropathological profile (39). While advanced dementia involves widespread neuronal degeneration, MCI patients maintain preserved neuroplasticity and functional capacity, rendering them particularly responsive to targeted cognitive stimulation (40). VR technology generates ecologically valid environments through multisensory integration and real-time interaction, effectively engaging neural networks underlying memory, attention, and executive functions (41, 42). Our findings, consistent with accumulating evidence (43–46) confirm that VR-based interventions significantly enhance cognitive performance in MCI patients. Notably no interference is accepted (47) and demonstrated VR efficacy in improving cognitive function in brain tumor patients, while Kim et al. (48) reported enhanced outcomes when combining VR with computer-based rehabilitation in stroke patients. These collective findings underscore VR transdiagnostic potential in cognitive rehabilitation, with MCI patients deriving particular advantage due to their retained neuroplasticity. Our findings indicate that VR-based games outperform structured cognitive training in rehabilitation efficacy, primarily attributable to their dual “entertainment-therapy” nature. By incorporating narrative tasks, reward mechanisms, and adaptive difficulty, these games effectively sustain engagement and overcome adherence limitations common in conventional training (10, 37). Empirical evidence confirms this advantage: Muñoz et al. (49) demonstrated that gamified VR tasks integrating motor-cognitive components significantly enhance participation, while Yanguas et al. (50) reported substantial cognitive improvements through VR gaming applications. Conversely, VR cognitive training employs structured protocols targeting specific domains, potentially yielding focused effects but lacking comparable motivational engagement. Although statistical significance was not achieved—possibly due to sample size constraints—the consistent effect pattern suggests clinical relevance for intervention selection.

Notably, the moderate heterogeneity observed in the study (*I^2^* = 64.69%) underscores the necessity of developing standardized intervention protocols. The findings of Moulaei et al. (11) further emphasize the importance of considering specific design elements—such as the immersive characteristics of virtual reality environments—for achieving positive outcomes. In exploring potential sources of this heterogeneity, a notable finding emerging from the meta-regression is that the level of VR immersion—ranging from low-cost head-mounted displays to fully immersive systems—significantly moderates intervention efficacy (*β* = 0.834, *p* < 0.05). Interpretation of hardware-based immersion in meta-regression requires distinguishing technical immersion (objective system attributes) from subjective presence (the psychological sense of “being there”). While technical immersion establishes the foundation for presence through multisensory integration, presence intensity remains equally dependent on content design and individual factors (51). This conceptual distinction clarifies that the benefits of high-immersion systems operate primarily through presence-mediated pathways. Soh et al.’s research (52) further corroborated the interference-shielding effect of immersion in remote virtual rehabilitation. As demonstrated by Torpil et al.’s (37), heightened hippocampal and prefrontal activation under high-immersion VR conditions suggests presence may enhance cognitive outcomes by reducing environmental interference and deepening emotional engagement. Our meta-regression, however, could only approximate these mechanisms indirectly through hardware specifications. Future investigations should directly quantify presence using standardized measures while examining its mediating role between technical parameters and cognitive outcomes. Concurrently, optimizing immersion through haptic feedback and 360° rendering must balance technological advancement with individual tolerance in elderly MCI populations. This integrated approach will advance our understanding of VR therapeutic mechanisms while ensuring clinical applicability.

GRADE evaluation confirms moderate-quality evidence supporting VR interventions for cognitive improvement in MCI, establishing them as valid non-pharmacological alternatives. However, VR-based gaming specifically demonstrates low evidence certainty, warranting exploratory application. Clinical implementation should align with care settings: structured task-based protocols in hospitals, engaging games in community centers, and portable device training for home use. All applications require personalization of duration, frequency, and difficulty based on individual patient profiles. As demonstrated by Samarasinghe et al. (53) in their development of VR games for Alzheimer’s patients, tailoring interventions to individual cognitive profiles is essential. Future development should focus on standardizing immersion metrics, creating age-appropriate interfaces, and establishing remote support systems for home-based VR training. These steps are essential for ensuring intervention consistency and accessibility. To strengthen the evidence base, future research should adhere to GRADE recommendations through large-scale RCTs with enhanced blinding procedures and comprehensive outcome reporting. Such methodological rigor will address current limitations in precision and heterogeneity, ultimately supporting the standardized integration of VR into cognitive rehabilitation protocols.

## 5 Limitations

This study has several limitations that should be considered when interpreting the findings. First, according to the GRADE assessment, the limited number of available trials and their aggregate sample size led to imprecise estimates, as reflected in wide confidence intervals. This imprecision was a key reason for the GRADE assessment of moderate (for overall VR efficacy) to low (for VR games) certainty of evidence. Second, the included studies predominantly featured short-term follow-up periods, which restricts our ability to draw firm conclusions regarding the long-term sustainability of the cognitive benefits derived from VR interventions. Third, the geographical distribution of the evidence is skewed, with 8 of the 11 included studies conducted in South Korea, potentially limiting the cross-cultural generalizability of the results. Fourth, the definition and measurement of “VR immersion level” were inconsistent across studies, challenging a standardized comparison of its moderating effect. Finally, the absence of double-blinding in all trials introduces a potential for performance and detection bias.

## 6 Conclusion

This systematic review of 11 randomized controlled trials establishes that VR-based cognitive training produces significant cognitive improvements in mild cognitive impairment, with technical immersion level serving as a crucial moderating factor. Achieving optimal outcomes requires balancing technological sophistication with individual cognitive adaptability to promote sustained engagement. To advance this field, future multi-center trials featuring extended follow-up periods and culturally diverse cohorts are essential for validating long-term efficacy and developing personalized intervention protocols.

## Data Availability

The original contributions presented in the study are included in the article/supplementary material, further inquiries can be directed to the corresponding author.
