# Correction: Effectiveness of VR-based cognitive training and games on cognitive rehabilitation in patients with MCI: a systematic review and meta-analysis

**DOI:** 10.3389/fneur.2025.1749179

**Published:** 2025-12-15

**Authors:** Peiming Yuan, Jiaxi Chen, Dianhui Peng, Qian Yang, Bin Liu, Chunxia Lu

**Affiliations:** College of Physical Education, Hunan Normal University, Changsha, China

**Keywords:** virtual reality, VR cognitive training, mild cognitive impairment, cognitive rehabilitation, games, meta-analysis

In the published article, author Chunxia Lu was erroneously omitted from the Correspondence section.

The correct corresponding authors should be Bin Liu and Chunxia Lu.

The original article has been updated.

